# Identification and Content of Astaxanthin and Its Esters from Microalgae *Haematococcus pluvialis* by HPLC-DAD and LC-QTOF-MS after Extraction with Various Solvents

**DOI:** 10.3390/plants10112413

**Published:** 2021-11-09

**Authors:** Biljana Todorović, Veno Jaša Grujić, Andreja Urbanek Krajnc, Roman Kranvogl, Jana Ambrožič-Dolinšek

**Affiliations:** 1 Department of Botany and Plant Physiology, Faculty of Agriculture and Life Sciences, University of Maribor, Pivola 10, SI-2311 Hoce, Slovenia; biljana.todorovic@um.si (B.T.); andreja.urbanek@um.si (A.U.K.); 2 Department of Biology, Faculty of Natural Sciences and Mathematics, University of Maribor, Koroška 160, SI-2000 Maribor, Slovenia; veno.grujic@um.si; 3 Department of Elementary Education, Faculty of Education, University of Maribor, Koroška 160, SI-2000 Maribor, Slovenia; 4 National Laboratory of Health, Environment and Food, Prvomajska 1, SI-2000 Maribor, Slovenia; roman.kranvogl@nlzoh.si

**Keywords:** astaxanthin diesters, astaxanthin monoesters, carotenoids, *Hematococcus pluvialis*, astaxanthin diesters, solvent extraction

## Abstract

*Haematococcus pluvialis*, a unicellular green microalga that produces a secondary metabolite under stress conditions, bears one of the most potent antioxidants, namely xanthophyll astaxanthin. The aim of our study was to determine the content of astaxanthin and its esterified forms using three different solvents—methyl *tert*-butyl ether (MTBE), hexane isopropanol (HEX -IPA) and acetone (ACE)—and to identify them by using high performance liquid chromatography coupled with diode array detection and the quadrupole time-of-flight mass spectrometry (HPLC-DAD and LC-QTOF-MS) technique. We identified eleven astaxanthin monoesters, which accounted for 78.8% of the total astaxanthin pool, six astaxanthin diesters (20.5% of total), while free astaxanthin represented the smallest fraction (0.7%). Astaxanthin monoesters (C16:2, C16:1, C16:0), which were the major bioactive compounds in the *H. pluvialis* samples studied, ranged from 10.2 to 11.8 mg g^−1^ DW. Astaxanthin diesters (C18:4/C18:3, C18:1/C18:3) were detected in the range between 2.3 and 2.6 mg g^−1^ DW. All three solvents were found to be effective for extraction, but MTBE and hexane-isopropanol extracted the greatest amount of free bioactive astaxanthin. Furthermore, MTBE extracted more low-chain astaxanthin monoesters (C16), and hexane-isopropanol extracted more long-chain monoesters (C18 and above) and more diesters. We can conclude that MTBE is the solvent of choice for the extraction of monoesters and hexane-isopropanol for diesters.

## 1. Introduction

Microalga *Hematococcus pluvialis* is a unicellular green microalga that produces the secondary metabolite xanthophilic astaxanthin, which accumulates under extreme stress conditions [1,2,3,4,5]. It is a highly potent antioxidant with antioxidant activity, which is ten times stronger than in the case of β-carotene [6] and 500 times stronger than in the case of α-tocopherol [7]. It is known for its ability to increase the in vivo activities of a series of antioxidant enzymes and to preserve the biological membrane structure [8]. Moreover, it has anti-inflammatory, anti-apoptotic, anti-aging, anti-cancer, anti-obesity, cardioprotective, immunomodulatory, anti-diabetic, hepatoprotective and neuroprotective properties and is even cytotoxic against gastric cancer cells [8,9,10,11]. Unlike some other carotenoids it has no pro-oxidant effect [8].

As a promising dietary supplement, astaxanthin has great potential for use in food, dietary supplements, cosmetics, agriculture, aquaculture, and medicine [9,12]. Moreover, natural astaxanthin from *H. pluvialis* has greater antioxidant properties than synthetic ones [3]. Therefore, research related to astaxanthin from natural sources has visibly attracted the scientific community and has great potential in terms of application.

In nature, it occurs in *H. pluvialis*, one of the most potent organisms for astaxanthin production, as well as in many other organisms, especially marine organisms [8,9,10,12]. Besides astaxanthin, *H. pluvialis* contains several other carotenoids, the predominant ones being β-carotene, α-carotene, β-cryptoxanthin, lycopene, lutein and violaxanthin, as well as a number of other bioactive compounds such as proteins, lipids and other bioactive substances [13,14].

The red phase of the alga *H. pluvialis* is considered the most valuable source of carotenoids, including a high content of astaxanthin, which is mostly present in bound lipid form as monoesters and diesters. Bound and unbound astaxanthin accumulates in the individual cells of microalgae, which are surrounded and protected by a solid envelope that must be disrupted before extraction. The resulting content of astaxanthin and other carotenoid substances depends on the type of solvent and the extraction method. Various extraction methods have been used for the efficient extraction of astaxanthin and other carotenoids. Several authors have used methods in which the encapsulated cells in the red phase were pretreated with organic and mineral acids at higher temperatures and with lytic and other enzymes releasing the constituents from cells with thick cell walls. This was followed by extraction with acetone or vegetable oils [15,16,17,18]. Some of the authors evaluated different extraction procedures by using acetone, a mixture of hexane and isopropanol (6:4, *v*/*v*), the extraction of methanol followed by two-step acetone and methanol extraction, and extraction with soybean oil [18]. Fábryová et al. [8] evaluated the biphasic extraction with ethanol/ethyl acetate. The authors state that the critical factors for maximum extraction are time, temperature and acid concentration.

Astaxanthin occurs naturally either in unesterified (yeast) or esterified form (algae). In *H. pluvialis* it is found mainly in esterified form as monoester bound to short-chain fatty acids [19,20,21]. Approximately 70% of the astaxanthin pool of *H. pluvialis* with enzyme formation are monoesters, 25% diesters, and 5% free astaxanthin, predominantly in 3S,3S’ form [22].

Astaxanthin has been quantified by various analytical methods, such as HPLC-DAD [8,12,16,18,21,23] and LC-(APCI)MS [24,25,26]. Castillo et al. [27] focused on environmentally friendly solid phase dispersion (MSPD) extraction with ethanol, ethyl lactate and water where they obtained three new acids, determined some astaxanthin monoesters and no diester. The, however, did not determine whether this MSPD method is suitable for identifying diesters and the large number of monoesters in extracted algae. Although there have been several attempts, a more detailed analysis of the lipid forms of mono- and diesters of astaxanthin is lacking.

The purpose of our study was to improve and optimize the efficiency of the extraction method for free and bound astaxanthin forms in the red phase of the alga *H. pluvialis.* Furthermore, the objective was to identify and quantify free and esterified forms of astaxanthin and its mono- and diesters by using HPLC-DAD and LC-QTOF-MS. In addition, we attempted to determine whether different solvents affect the content and composition of individual astaxanthin derivatives.

## 2. Results

### 2.1. Free and Esterified Forms of Astaxanthin

**Figure 1:**
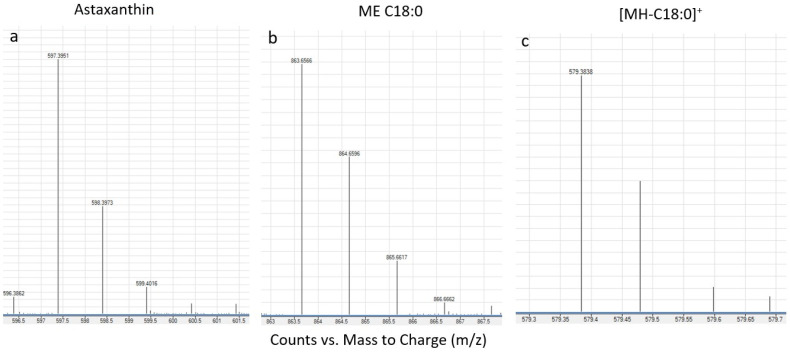
LC-QTOF-MS mass spectra (ESI, positive ion mode) of (**a**) astaxanthin, (**b**) astaxanthin monoester [ME C18:0] and (**c**) *m/z* 579.3840 [MH-C18:0]^+^.

The analyses performed by using LC-QTOF-MS have better sensitivity and selectivity and are more accurate compared to those performed by HPLC-DAD. The accuracy of the bioactive substances by LC-QTOF-MS was determined on the basis of molecular weight. Analysis of the samples in the case of LC-QTOF-MS revealed 15 astaxanthin monoesters, 11 diesters and a free form of astaxanthin. Among them, 11 astaxanthin monoesters and six astaxanthin diesters were identified with their molecular weights (Table 1 and Table 2). The high resolution *m/z* 597.3938 and 579.3840 are protonated quasimolecular ions [M + H]^+^ and [MH-18]^+^ of astaxanthin, based on the mass spectra in contrast and positive ion modes, respectively. The large amounts of astaxanthin fatty acid monoesters (ME) were assigned as (ME + H)^+^ and their typical fragmentation products (M-FA)^+^. The *m/z* 863.6554 represents [M + H]^+^ of ME-C18:0. The 579.3840 appears to be [MH-C18:0]^+^ (Figure 1). Astaxanthin fatty acid diesters (DE) were also determined in terms of their *m/z* (DE+H) and their fragments. The identification was confirmed by the accurate high resolution mass (<5 ppm) of adducts, fragmentation products (M-FA) and their retention time (Rt window for ME (31.5–42 min) and Rt window for DE (41.5–45 min)). The location of the double bonds could not be unambiguously identified because mass differences between quasimolecular and fragment ions were used to assign the acyl chains. We observed that the fragmentation pattern of astaxanthin esters was dominated by the loss of fatty acid. Long-chain fatty acids with 16 or 18 carbon atoms in the forms C16:0, C16:1, C16:2, C18:1, C18:2, C18:3, and C18:4, as well as C20:0 or C26:2, predominated. Most of them, namely 78.8%, were monoesters, 20.5% diesters, and only 0.7% of free astaxanthin were determined (Table 1 and Table 2).

Astaxanthin monoesters (ME) were the major constituent of the *H. pluvialis* samples. Their detected contents ranged from 0.01–11.8 mg g^−1^ DW. The maximum content of the ME-C16:1 sample was 11.8 mg g^−1^ DW and the maximum contents of the ME-C16:0 and the ME-C16:2 samples were 10.2 mg g^−1^ DW. All other contents were lower. Long-chain fatty acids with 20 carbon atoms (ME-C20:0 and ME-C20:1) were identified as monoesters (Table 2).

Astaxanthin diesters (DE) ranged from 0.05–2.7 mg g^−1^ DW. The maximum content of the DE C18:4/C18:3 sample was 2.7 mg g^−1^ DW. The free form of astaxanthin was detected in the lowest content, namely 0.2–0.3 mg g^−1^ DW (Table 2).

Four of the 15 detected monoesters and three of the 11 detected diesters were not identified. The unidentified monoesters reached a maximum level of 2.2 mg g^−1^ DW while the unidentified diesters reached 0.5 mg g^−1^ DW (Table 2). Two monoesters were identified, namely (C18:0-C18:1), but we could not determine their individual molecular weight (Table 2). Six diesters were detected in the LC-QTOF-MS spectral region, three of which were identified as C26:1-C26:2, C18:2-C18:3 a and C18:2-C18:3 b (Table 2), while three diesters were not identified (DE-nd 1, DE-nd 2 and DE-nd 3) (Table 2).

### 2.2. The Effect of the Solvent

Three different extraction solvents were compared, that is, those releasing carotenoids from the cells in the surrounding solvents: MTBE, HEX-IPA and ACE after HCl pretreatment (70 °C for 5 min).

The total content of astaxanthin (free and esterified forms) was the highest when MTBE was used, yielding an average of 54.0 mg g^−1^ DW of total astaxanthin (free and esterified forms) and, out of these, there is an average of 44.8 mg g^−1^ DW of monoesters, 8.8 mg g^−1^ DW of diesters and 0.3 mg g^−1^ DW of free astaxanthin.

The solvent HEX-IPA extracted an average of 54.5 mg g^−1^ DW of the total astaxanthin pool and, of these, an average of 44.7 mg g^−1^ DW of monoesters, 9.5 mg g^−1^ DW of diesters and 0.3 mg g^−1^ DW of free astaxanthin. The solvent ACE extracted an average of 28.3 mg g^−1^ DW of the total free and esterified forms of astaxanthin and of these, an average of 23.2 mg g^−1^ DW of monoesters, 4.9 mg g^−1^ DW of diesters and 0.2 mg g^−1^ DW of free astaxanthin. The content of free astaxanthin extracted with solvents MTBE and HEX-IPA (0.3 mg g^−1^ DW) is 1.5 times higher than when solvent ACE was used with a content of 0.2 mg g^−1^ DW (Table 2).

**Figure 2:**
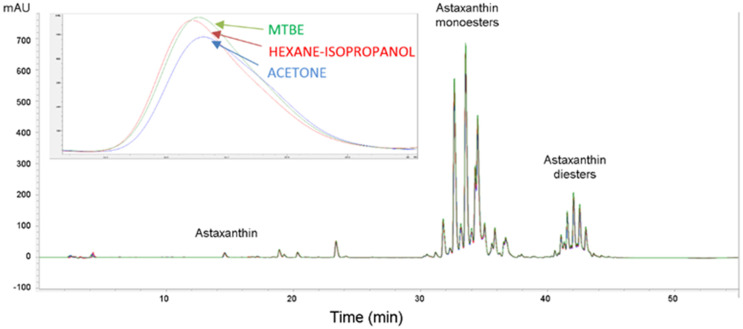
HPLC chromatogram (DAD, 475 nm) astaxanthin, mono− and diesters in three different solvents (MTBE: methyl *tert*-butyl ether, HEX-IPA: hexane-isopropanol; ACE: acetone). The inserted picture shows the enlarged free astaxanthin chromatogram. Solvents MTBE and HEX-IPA extracted a significantly higher amount of free astaxanthin.

**Figure 3:**
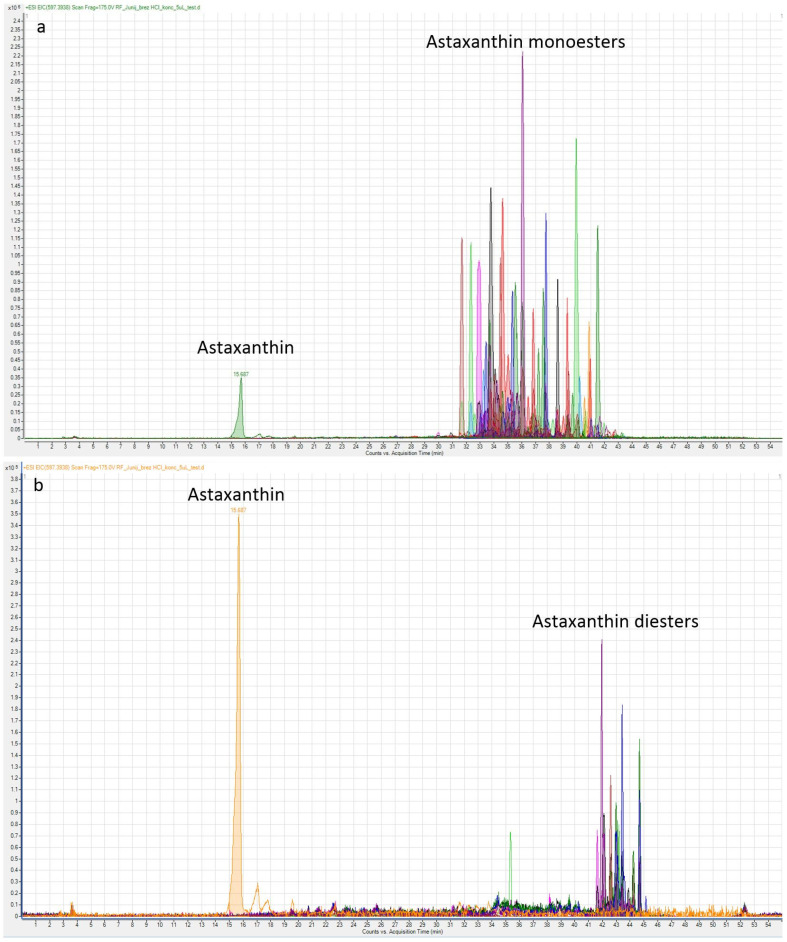
LC-QTOF-MS chromatograms, astaxanthin, (**a**), monoesters and (**b**), diesters.

The content of monoester ME-C16:0 extracted with solvent MTBE was 2.3 times higher (10.2 mg g^−1^ DW) and with HEX-IPA (9.7 mg g^−1^ DW) it was 2.2 times higher than when solvent ACE was used (Table 2). The content of ME-C16:2 was on average 1.8 times higher when using the solvent MTBE (10.2 mg g^−1^ DW) and the solvent HEX-IPA (10.2 mg g^−1^ DW) compared to using ACE with a content of 5.6 mg g^−1^ DW. The content of ME C16:1 extracted with solvents MTBE and HEX-IPA (11.8 mg g^−1^ DW) was 1.9 times higher than that regarding the solvent ACE with a content of 6.3 mg g^−1^ DW (Table 2). The same was found for diester, as the content of DE-C18:4/C18:3 was on average 2.1 times higher when the solvents MTBE (2.6 mg g^−1^ DW) and HEX-IPA (2.7 mg g^−1^ DW) were used, compared to the use of ACE with a content of 1.6 mg g^−1^ DW. All three solvents extracted most of the astaxanthin and its esters (Table 2, Figure 2 and Figure 3a,b). However, solvents MTBE and HEX-IPA provided significantly higher amounts of astaxanthin and its esters than solvent ACE.

Solvent HEX-IPA was more effective than MTBE in extracting long chain monoester ME-C18:4, and its content was 1.3 times higher (1.1 mg g^−1^ DW) compared to extraction with solvent MTBE. Solvent HEX-IPA was more efficient in the extraction of diesters compared to MTBE, especially in the case of long chain diester DE-C18:2/C18:3 b (Table 2). Moreover, solvent HEX-IPA provided a higher content of the minor components of the extract, the monoesters with longer chains ME-C18:4, ME-C18:3 and ME-C18:2 than solvent MTBE.

There is also no statistically significant difference in the content of some long chain monoesters (C20:2, C20:1 and C20:0) relating to the solvents MTBE and HEX-IPA. However, there is a statistically significant difference in the content of long chain monoesters relating to extraction with MTBE and ACE, and with HEX-IPA and ACE (Table 2). For example, the content of C20:2 monoester extracted with MTBE is on average 2.0 times higher than the content of this monoester extracted with ACE. The content of C20:1 monoester extracted with HEX-IPA solvent was 1.9 times higher than the content of the same monoester in the ACE solvent (Table 2). The extraction efficiency for diesters is similar to that for monoesters (Table 2). MTBE and HEX-IPA were much more efficient as solvents than ACE, except for DE-C18:2/C18:3 b, where ACE proved to be an effective solvent and DE-C18:2/C18:3 a, indicating a statistically significant difference (Table 2). In the LC–MS analysis we could not identify three diesters (Table 2). In the case the first diester (DE-nd 1), we found that HEX-IPA and ACE proved to be the better solvent in terms of content, but there was no statistically significant difference. The results for the diester (DE-nd 3) showed that solvents MTBE and HEX-IPA were more efficient than the solvent ACE in terms of content.

**Figure 4:**
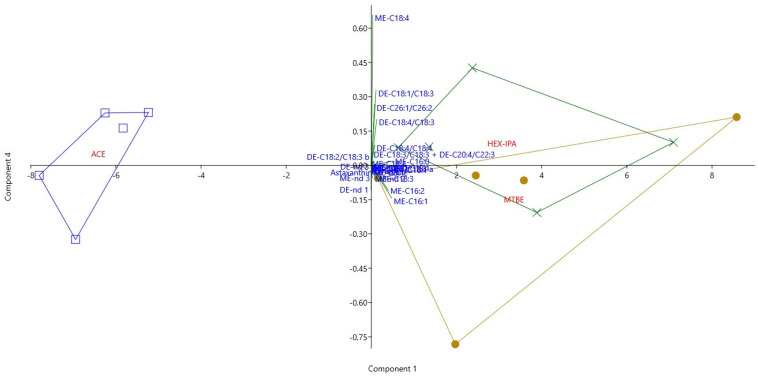
Principal component diagram (PCA) of astaxanthin derivatives (free astaxanthin, astaxanthin monoesters−ME and diesters−DE) in relation to different extraction solvents: ACE (blue square), HEX-IPA (green cross) and MTBE (dark golden dot). For detailed statistics, see Table 3.

In order to provide a comprehensive assessment of individual astaxanthin derivatives and to further examine the extraction efficiency of the various solvents, we performed a principal component analysis. Principal component 1 (PC1) accounts for 96.507% of the variance and is most strongly correlated with the predominant short-chain monosters (ME-C16:2, ME-C16:1, ME-C16:0). The fourth PC accounts for 0.293% variance and is positively correlated with ME C18:4 and predominant diesters (DE-C18:4/C18:4; DE-C18:4/C18:3, DE-C18:1/C18:3, DE-C26:1/C26:2), while negatively correlated with short-chain monoesters (ME-C16:2, ME-C16:1) and diesters DE-nd 1 (Table 3). The results of the PCA analysis are shown graphically in Figure 4. The first function discriminates probes extracted with ACE solvent in negative direction from other having clearly smallest concentrations of analyzed astaxanthin derivatives except for DE-C18:2/C18:3 b. In addition, PC4 separates samples extracted, with HEX-IPA being more efficient in the extraction of ME C18:4 and diesters from samples extracted with MTBE, which proved to be more effective in the extraction of short chain monoesters (Table 3, Figure 4).

## 3. Discussion

A thick and rigid cell wall of red, immobile palmelloid cells filled with carotenoids was the target of a pretreatment for the extraction of astaxanthin. Chemical pretreatment of the cell walls with HCl allowed the release of carotenoids from the cells into the surrounding solvents [16,18]. Sarada et al. [16] stated that a concentration of 1–2 N HCl with 5–10 min heating at 70 °C resulted in 96% extraction efficiency, visible under the microscope as colorless cells with intact cell walls. In our experiments, cells were completely decolorized with all three solvents, which was confirmed with observation under microscope. Chemical pretreatment of the cell walls with HCl is a good and inexpensive way to release carotenoids from the cells without mechanical treatment, although this method has been reported to result in significant losses [28].

### 3.1. Free and Esterified Forms of Astaxanthine

In addition to the promotion, efficiency, and production costs of *H. pluvialis* [8], the highlighted current research objectives were the isolation of astaxanthin and the separation and determination of the free and esterified forms of astaxanthin. Our studies revealed that very little astaxanthin isolated from *H. pluvialis* existed in free form. Most of it was in the form of monoesters and less in the form of diesters. Our analysis of astaxanthin content is consistent with that of Sarada et al. [16] who found that astaxanthin of the microalga *H. pluvialis* consisted of 72.0% of monoesters, 27.5% of diesters, and 0.5% of free astaxanthin. The authors’ results are comparable to the composition of our samples, which contained 78.8% of monoesters, 20.5% of diesters, and 0.7% of free astaxanthin. In addition, our results are comparable and in agreement with the results of Peng et al. [29], who analyzed several different strains of algae and found that the *H. pluvialis* alga consisted of 60.9% monoester and 35.5% diester content. According to the average total expressed astaxanthin content (mg g^−1^), our results were 54.0 mg g^−1^ DW when extracted with solvent MTBE, 54.5 mg g^−1^ DW when extracted with solvent HEX-IPA and 28.3 mg g^−1^ DW when extracted with solvent ACE. The average total astaxanthin content was significantly higher than the average astaxanthin content determined by Peng et al. [29] who determined 16.1 mg g^−1^ of total astaxanthin content on average with an average of 9.8 mg g^−1^ of monoester, 5.7 mg g^−1^ of diester and 0.6 mg g^−1^ of free astaxanthin extracted with the mixture of ethanol and dichloromethane (75:25, *v*/*v*).

Although the health-promoting properties and safety profile of astaxanthin in free form have been extensively studied and recognized, esterified astaxanthin may be responsible for the biological properties attributed to astaxanthin [16]. Astaxanthin esters have higher thermal stability and greater bioavailability than astaxanthin in free form [30]. Most of the astaxanthins in our samples were in bound form, which means that they are more thermostable and have greater biological potential than free forms of astaxanthin. Holtin et al. [26] reported that the main components of the complex astaxanthin extract were separated by HPLC and the main compound astaxanthin ester was formed by different fatty acids. They pointed out that the most abundant fatty acids were defined as (C18:3, C18:2, C18:1 and C16:0). This is in agreement with our results.

### 3.2. Extraction Solvents

Zhou et al. [30] dissolved astaxanthin esters from algae *H. pluvialis* in methanol/methyl *tert*-butyl ether (MTBE) (1; 1, *v*/*v*)) and separated them by HPLC-(+) APCI/MS. They analyzed and identified twenty astaxanthin esters, eight of them being monoesters and twelve diesters. They indicated fatty acids with 16 or more carbon atoms (C18:1, C18:2, C18:3, C18:4, C16:0 and C16:1). As for the total astaxanthin, about 85% represent monoesters and 15% diesters [30]. This means that monoesters are the most abundant form of astaxanthin esters, which again is consistent with our results.

The main monoesters of the microalga *H. pluvialis* clearly contain acyl chains C18:3 (2), C18:2 (3), C18:1 (4), and C16:0 (5) [31]. The same authors, namely [31], explain that the degradation of astaxanthin esters and the formation of fragmented ions are due to the loss of one or two fatty acids. Our results, too, show that the loss of fatty acids predominates in the pattern of fragmented astaxanthin esters.

In the LC/ESI-MS analysis, Frassanito et al. [32] observed a characteristic loss of the carotenoid units from the conjugated polyene part. They noted that important ions are not observed in the APCI (atmospheric pressure chemical ionization) mass spectrum with positive and negative ions. In addition to the detected ions, the ESI-MS/MS positive and/or negative ions did not identify the main structural features of the acyl chains (length, position and saturation), but indicated nominal mass of the parent xanthophyll. Our results also show exact molecular weights for eleven monoesters, six diesters and free astaxanthin with no artificial loss from the conjugated polyene part of the carotenoid unit.

With regards to LC-(APCI) MS analysis Miao et al. [24] determined four free carotenoids, fifteen astaxanthin monoesters, twelve astaxanthin diesters and three astacin monoesters. The identification of each compound was based on the characteristic fragment ions of the positive ion, negative ion mode, and MS (2). They also reported on astaxanthin esters resulting from the loss of one or two fatty acids. These are also in agreement with our results. The authors pointed out that astaxanthin monoesters in the positive ion mode exhibited characteristic fragmented ions at m/z 597 [M + H -fatty acid]+ and *m/z* 579 and 561, which is due to constant water loss. The same authors explain that the relative *m/z* intensity 579 in MS2 was more than 80% of the molecular ion intensity. In diesters, the intensity *m/z* 561 was occasionally the same as m/z 579, but in general the former, accounting for 50% to 60% or more of the molecular ion, was stronger than the latter, which decreased by 20% to 30% of the molecular ion. Characteristic fragment ions were detected at *m/z* 871 and m/z 593 in the positive MS2 ion mode [24]. This is also consistent with our results. The same authors note that in liquid chromatography analysis, many bioactive compounds show the highest adsorption at 400 nm.

In the HPLC-DAD analysis, the absorbance and the determination of astaxanthin in *H. pluvialis* was measured at 444–450–478 nm [23]. Sarada et al. [16] determined a characteristic peak at 475 nm and a non-characteristic peak at 645–660 nm, indicating the absence or trace of the nm range of chlorophyll and this is also consistent with our results. Peng et al. [29] also determined a characteristic peak at 480 nm after checking the spectral range between 300 and 650 nm, which again is consistent.

The use of MTBE and HEX-IPA solvents is not recommended in industry, especially when astaxanthin and other carotenoids are used in the food, pharmaceutical or cosmetic industries. Our study compares them with more frequently used ACE and focuses on the identification of astaxanthine and its esters for research purposes.

All three different solvents were found to be efficient for the extraction of astaxanthin and its esters. However, we found that MTBE and HEX-IPA were significantly more effective than the solvent ACE. Based on PCA analysis, solvent MTBE proved to be more effective in the identification of short chain monoesters and HEX-IPA proved to be a suitable solvent for the analysis and identification of long chain monoesters and diesters.

MTBE was also the solvent of choice in the research by Bauer and Minceva [33], who used direct liquid–liquid extraction of astaxanthin from *H. pluvialis* red phase. The same authors explain that a hard cell wall prevents direct extraction of a dye from a cell. They tested several solvents for the extraction of astaxanthin, namely ethyl acetate (EtOAc), MTBE, dichlormethane (DCM) and butan-1-ol. Finally, MTBE was chosen, which proved to be very effective in liquid chromatography. Bauer and Minceva [33] pointed out that it was not possible to keep the stationary phase in the chromatography column with the green solvent ethyl acetate (EtOAc). Although they tested the environmentally friendly solvent EtOAc, they finally used MTBE for further extraction experiments. They emphasize that this could be a concept for an alternative process. The results of the authors Bauer and Minceva [33] are consistent with our results regarding the suitability of the solvent MTBE in liquid chromatography (in the separation and identification of astaxanthin). Our analyses employ MTBE to charge the mobile phases, and this proved to be effective.

Our choice of the more effective solvents is not in agreement with results of Dong et.al. [18], who tested four extraction solvents (ACE, HEX-IPA, methanol-acetone (MET-ACE), soybean oil) and found solvent ACE to be the most effective among them. In our study, ACE was the slightly more effective solvent only for some low amount diesters DE-C18:2/C18:3a and DE-C18:2/C18:3b.

Some authors [23] have studied the effect of time, pressure, temperature, and mechanical pretreatment on astaxanthin extraction using and comparing GRAS with a more toxic solvent. Astaxanthin excretion was very poor in the first batch (less than 1%) regardless of solvent or operating conditions. In the seconds series, they achieved a very high yield of astaxanthin by mechanical pre-treatment. Otherwise, ACE extraction of astaxanthin was found to be 87% effective for one hour at 10 Mpa and at 40 °C [23]. The same authors [23] point out that, after mechanical pre-treatment, a single extraction procedure of 20 min is sufficient to extract more than 99% of the total extracted astaxanthin [23].

Our analyses confirm the results of other studies [8,16,22,29] in that the alga *H. pluvialis* contains a very small amount of free astaxanthin. Most of the astaxanthin is synthesized by the alga in bound form (monoesters and diesters). Since these are esters (lipid forms), we chose non-polar solvents (MTBE and HEX-IPA) for appropriate and successful identification and quantification, and compared them to the widely used and a more environmentally friendly solvent ACE.

The solvent MTBE proved to be effective in the extraction and determination of astaxanthin content. When filling up the mobile phase (liquid chromatography), the solvent MTBE was the most suitable for the separation and identification of astaxanthin and its esters.

## 4. Materials and Methods

### 4.1. Microalgae Culture

The microalga *H. pluvialis* used in our experiments was provided by Autotrophic Organisms Collection (CCALA), Trebon Czech Republic. Axenic *H. pluvialis* strain CCALA 840 was transferred from agar stock cultures into 250 mL of Bold’s Basal Medium (BBM) [34,35]. After 14 days, the algal suspension was inoculated into 15 Erlenmeyer flasks containing 250 mL of BBM at an initial algal density of 20 mg L^−1^. In our experiments, immobile red palmelloid algal cells surrounded by a solid cell wall were used after 30 days of single-stage cultivation.

Culture conditions were 25 ± 1 °C, 16 h/8 h light/dark cycle at a light intensity of 140 μmol photons m^−2^ s^−1^, illuminated from below. Cultures were aerated with sterile humidified air (1.85 L air per L^−1^ culture medium min^−1^) with the addition of 1.5% carbon dioxide (CO2) and shaken twice daily. To check the purity of the algal cultures, a solid medium was prepared with an additional 1% of peptose, glucose and yeast extract. The algal cultures were considered axenic when no bacterial colonies developed on the solidified plate after 7 days of incubation at 25 ± 2 °C [36].

### 4.2. Dry Weight Determination

Dry weight (DW) of microalgal biomass was determined on the basis of the differences in dry weight before and after filtering the algal suspension. The microalgae werecollected on Whatman GF/C glass fiber filters, first dried at 105 ± 5 °C for 3 h, cooled in a desiccator, weighed, and used to filter 10 mL of the microalgae samples. The filters containing the microalgae suspension were again dried at 105 ± 5 °C for 3 h, cooled in a desiccator, and weighed. The density of the suspension was calculated as mg L^−1^ DW.

### 4.3. Hydrochloric Acid Carotenoid Extraction

Hydrochloric acid (HCl) chemical pretreatment of algal cell walls allowed the release of carotenoids from the cells into the surrounding solvents. Ticks and rigid cell walls of algal cells were disrupted with HCl. Samples of 5 mL of algal solution were centrifuged at 4000 rpm for 5 min and the supernatant was removed. Pellet of algal cells were pretreated with 2 mL of 1 M HCl at 70 °C for 2 min in a heating block [16], cooled, and centrifuged at 4000 rpm for 5 min. The supernatant was removed, and the sediments were washed twice with 2 mL of deionized water. The remaining pellet of the algal cells was prepared for extraction and estimation of carotenoids by using three different solvents: tert-butyl methyl ether (MTBE) from Fluka Chemie (Buchs, Switzerland), hexane-isopropanol (HEX-IPA) (6:4 vol) from Carlo Erba (Italy) or Merck (Kenilworth, NJ, USA), and acetone (ACE) from Honeywell (Charlotte, NC, USA) with an additional 1% of butylated hydroxytoluene (BHT) to prevent oxidation of the carotenoids. Carotenoids from microalgal pellets were extracted into centrifuge tubes by adding 5 mL of solvent and 0.4 mm diameter glass beads, vortexed for 5 min, and centrifuged at 3000 rpm for 3 min. The extraction was repeated at room temperature until the sediment was completely colorless, but for no longer that 20 min. The extraction solvents and the whole process were carried out in the dark. The same procedures were performed for all three solvents. All extracts were filtered and stored in dark vials at −20 °C prior to analyses. We additionally checked the color of the disrupted cells under a light microscope for all three solvents. The supernatant was collected and stored in a dark place. We performed five replicates for each solvent.

### 4.4. HPLC-DAD and LC-QTOF-MS Analysis of Carotenoid Substances

Analyses were performed at the National Laboratory of Health, Environment and Food (NLZOH), Maribor. Astaxanthin and other bioactive carotenoid substances were separated using an Agilent 1260 HPLC system (Agilent technology, Santa Clara, CA, USA) coupled with a DAD detector. The spectrum was recorded from 200 to 800 nm. The wavelength for quantitative parameters was 475 nm. The column was Infinity Lab Poroshell 120 EC-C18: 150 × 3.0 mm, 2.7 µm, PN: 693975-302(T), SN: USCFW11265; the column temperature was 30 °C, the injection volume was 10.00 µL, the analysis time was 55 min. The method was modified according to Hrvolová et al. [37]. Mobile phases (A): 0.1% (*v*/*v*) formic acid in 50% ultrapure water and 50% methanol; (B): 0.1% (*v*/*v*) formic acid in 80% methyl *tert*-butyl ether and 20% methanol. The eluent flow was 0.2 mL min^−1^. The gradient started at 35% eluent B, changed to 90% eluent B over 40 min, held for 4 min and returned back to 35% eluent B. Equilibration lasted for 10 min. Processing Spectral data were processed using Open Lab software. Substance identification was performed in several steps, and was confirmed by comparing retention times with standard solution and spectral properties of the substance. The content of astaxanthin and other carotenoid substances was expressed in mg g^−1^ DW.

The individual bioactive compound identification was performed by using an Agilent 6530 LC-QTOF mass spectrometer with electrospray ionization (ESI) set to positive ionization. The compounds were analyzed over the entire mass range (from 50–1700 *m/z*). The chromatographic conditions and column settings were the same as for the HPLC-DAD analysis. The sample injection volume was 10.00 µL and the flow rate was 0.2 mL min^−1^. MS parameters were 4 GHz, high resolution was a maximum of 1700 *m/z*, acquisition rate 1.0 spectra/sec. Sample ionization was Dual ESI, and ion source positive ion scan mode used mass scanning from 50 to 1700 *m/z*. Other LC- QTOF parameters: drying gas (N2) and temperature 300 °C; drying gas flow rate 10 L/min; nebulizer 40 psig; Vcap. 4000 V; nozzle 2000 V; skimmer 65 V; fragmentor 175 V and Octopole RF 750 V. Spectral data were processed by using quality Agilent Mass Hunter Qualitative Software and Agilent PCDL Manager. Substance identification was performed by a comparison of retention times with standard solutions, the determination of accurate mass and the fragmentation of compounds. The contents of bioactive substances (carotenes) were expressed in mg g^−1^ DW. HPLC-grade methanol (J. T. Baker, Phillipsburg, NJ, USA) and MTBE were purchased from Sigma Aldrich, USA. The formic acid (≥99%) for LC-MS was purchased from VWR Chemicals (Chicago, IL, USA). Astaxanthin (≥97%) was purchased from Sigma Aldrich (Saint Louis, MO, USA), whereas astaxanthin esters come from *Haematococcus*, USP Reference standard. The remaining chemicals were of analytical grade obtained from Sigma Aldrich (Saint Louis, MO, USA). Ultrapure water was used to prepare all solutions and mobile phases.

### 4.5. Statistical Analysis

Results are presented as means (n = 5) and standard errors (mean ± SE) and were statistically analyzed by one-way analysis of variance (ANOVA) using SPSS^®^ 27 software (SPSS Inc., Chicago, IL, USA). The level of statistical significance (*p*) between the different treatments was determined by the post-hoc Duncan test. Differences at *p* ≤ 0.05 were considered statistically significant. Each treatment was repeated five times

To obtain a comprehensive assessment of the effects of the extraction solvents and to closer define the correlative relationship between individual astaxanthin derivatives, we conducted a principal component analysis using the Past 4.03 (Hammer, 2020) software (Table 3, Figure 4). Principal component 1 (PC1) explains 96.507% of the variance, Principal component 4 (PC4) explains 0.293% of the variance (Table 3, Figure 4)).

## 5. Conclusions

A selected strain of *H. pluvialis* obtained from the Collection of Autotrophic Organisms in Culture (CCALA), Trebon, Czech Republic has not been previously analyzed for the carotenoid profile (astaxanthin, its monoesters and diesters). We found that our extracts contain 78. 8% of monoester, 20.5% of diester and 07% of free astaxanthin. The availability of the results for the public is particularly important for the scientific community to be able to compare the studied strains in further research. In addition, the study will provide useful information for companies looking for suitable strains for the potential industrial production of astaxanthin and other carotenoids.

Our research revealed that free astaxanthin and its esters were most effectively extracted with MTBE that also corresponded to the mobile phase charger of the HPLC-DAD and the LC -QTOF-MS analyses.

The combination of the HPLC-DAD analyses and sensitivity and selectivity of the LC-QTOF-MS allowed the identification of 15 monoesters and 11 diesters of astaxanthin in the analyzed extracts. The high sensitivity of the LC-QTOF-MS allowed the dispersion, proper ionization and decomposition of the identified bioactive substances into fragmentation products (M-FA)^+^.

In the content of some long-chain esters (monoesters and diesters), we determined HEX -IPA as having the best effect on extraction. Knowing the extraction potential of different solvents is useful for the prediction of the extraction process in the extraction yield. The biological properties of astaxanthin are popular in pharmacology and the food industry, while the *H. pluvialis* alga is a rich source of astaxanthin.

Our results will, therefore, be useful for selecting the most appropriate solvent for further research and identification of *H. pluvialis* algal carotenoids, and in promoting production practices in smaller markets.

## Figures and Tables

**Table 1:** Mass spectrometry data of astaxanthin and individual esterified astaxanthins in the extract.

No	*m/z*	Adduct	Production (M-FA)	Identification
1	597.3938	[M + H]^+^		Astaxanthin
2	831.5928	[ME + H]^+^	579.3840	ME-C16:2
3	833.6084	[ME + H]^+^	579.3840	ME-C16:1
4	835.6241	[ME + H]^+^	579.3840	ME-C16:0
5	855.5928	[ME + H]^+^	579.3840	ME-C18:4
6	857.6084	[ME + H]^+^	579.3840	ME-C18:3
7	859.6241	[ME + H]^+^	579.3840	ME-C18:2
8	861.6397	[ME + H]^+^	579.3840	ME-C18:1
9	863.6554	[ME + H]^+^	579.3840	ME-C18:0
10	883.6241	[ME + H]^+^	579.3840	ME-C20:2
11	885.6397	[ME + H]^+^	579.3840	ME-C20:1
12	887.6554	[ME + H]^+^	579.3840	ME-C20:0
13	1113.7906	[DE + H]^+^	860.6783	DE-C18:4/C18:4
14	1115.8062	[DE + H]^+^	862.6959	DE-C18:4/C18:3
15	1117.8219	[DE + H]^+^	870.7575	DE-C18:3/C18:3
16	1119.8375	[DE + H]^+^	872.7743	DE-C18:2/C18:3
17	1121.8532	[DE + H]^+^	874.7896	DE-C18:1/C18:3
18	1125.8845	[DE + H]^+^	888.7703	DE-C18:1/C18:1

ME: astaxanthin monoester; DE: astaxanthin diester; *m/z*: ratio of an ion’s mass (m) in atomic mass units (z) to its formal charge (z); M: molecule with molecular weight m; Product ion (M-FA); Adduct: product of a direct. Mass accuracy was less than 5 ppm.

**Table 2:** Content of all detected astaxanthin, mono- and di- esters (mean ± SE, mg g^−1^ DW) in *H. pluvialis* microalgae treated with different solvents.

No.	Compound	Content		
		Methyl *tert*-Butyl Ether (MTBE)	Hexane/Isopropanol (HEX-IPA)	Acetone (ACE)
1	Astaxanthin	0.31 ± 0.017 ^a,^*	0.30 ± 0.011 ^a^	0.20 ± 0.007 ^b^
2	ME-nd 1	0.32 ± 0.019 ^a,^*	0.32 ± 0.014 ^a^	0.18 ±0.006 ^b^
3	ME-nd 2	2.17 ±0.138 ^a^	2.15 ± 0.100 ^a^	1.19± 0.049 ^b^
4	ME-C16:2	10.17 ± 0.656 ^a^	10.17 ± 0.483 ^a^	5.56± 0.237 ^b^
5	ME-nd 3	0.28 ± 0.017 ^a^	0.24 ± 0014 ^a^	0.02 ± 0.0004 ^b^
6	ME-C18:4	0.82 ± 0.211 ^a,b^	1.10 ± 0.060 a	0.64 ± 0.033 ^b^
7	ME-C16:1	11.82 ± 0.769 ^a^	11.77 ± 0.589 ^a^	6.31 ± 0.266 ^b^
8	ME-C18:3	3.53 ± 0.152 ^a^	3.57 ±0.208 ^a^	1.85 ± 0.088 ^b^
9	ME-C16:0	10.20 ± 0.953 ^a^	9.70 ± 0.918 ^a^	4.41 ± 0.163 ^b^
10	ME-C18:2	0.13 ± 0.008 ^b^	0.19 ± 0.007 ^a^	0.10 ± 0.003 ^c^
11	ME-C20:2	0.97 ± 0.067 ^a^	0.99 ± 0.060 ^a^	0.48 ± 0.028 ^b^
12	ME-C18:0/C18:1	0.73 ± 0.043 ^a^	0.75 ± 0.035 ^a^	0.43± 0.027 ^b^
13	ME-C20:1	1.47± 0.097 ^a^	1.50 ± 0.073 ^a^	0.80 ± 0.042 ^b^
14	ME-C20:0	0.64 ± 0.034 ^a^	0.67 ± 0.042 ^a^	0.36 ± 0.020 ^b^
15	ME-nd 4	1.59± 0.114 ^a^	1.61± 0.077 ^a^	0.86± 0.047 ^b^
	**Total ME**	**44.84 ± 0.82**	**44.73 ± 0.80**	**23.20 ± 0.41**
16	DE-nd 1	0.15 ± 0.010 ^a,^*	0.50 ± 0.329 ^a^	0.53 ± 0.204 ^a^
17	DE-C18:4/C18:4	1.68 ± 0.124 ^a^	1.76 ± 0.089 ^a^	0.80 ± 0.098 ^b^
18	DE-C18:1/C18:1	0.13 ± 0.018 ^a^	0.12 ± 0.006 ^a^	0.06 ± 0.005 ^b^
19	DE-C18:4/C18:3	2.64 ± 0.203 ^a^	2.71 ± 0.149 ^a^	1.26 ± 0.166 ^b^
20	DE-C18:3/C18:3 + DE-C20:4/C22:3	0.20 ± 0.0210 ^a^	0.22 ± 0.0161 ^a^	0.13 ± 0.0167 ^b^
21	DE-C18:1/C18:3	2.34 ± 0.218 ^a^	2.41 ± 0.144 ^a^	1.17 ± 0.172 ^b^
22	DE-C26:1/C26:2	1.58 ± 0.157 ^a^	1.62± 1028 ^a^	0.79 ± 1264 ^b^
23	DE-C18:2/C18:3 a	0.03 ± 0.006 ^a,b^	0.02 ± 0.000 ^b^	0.05 ± 0.010 ^a^
24	DE-C18:2/C18:3 b	0.00 ± 0.000 ^c^	0.07 ± 0.003 ^a^	0.04 ± 0.005 ^b^
25	DE-nd 2	0.03 ± 0.001 ^b^	0.03 ± 0.001 ^b^	0.03 ± 0.004 ^b^
26	DE-nd 3	0.02 ± 0.000 ^a^	0.02 ± 0.000 ^a^	0.00 ± 0.000 ^b^
	**Total DE**	**8.80 ± 0.20**	**9.48 ± 0.20**	**4.87 ± 0.10**
	**Total Free astaxanthin**	**0.31 ± 0.008**	**0.30 ± 0.005**	**0.20 ± 0.003**
	**Total (free + ester forms)**	**53.97 ± 0.66**	**54.52 ± 0.65**	**28.26 ± 0.33**

* Different letters indicate statistically significant differences at *p* < 0.05. Values followed by the same letter do not significantly differ (ANOVA, Duncan post-hoc test, *p* < 0.05). ME: astaxanthin monoester; DE: astaxanthin diester; nd: not defined; MTBE: methyl *tert*-butyl ether; HEX-IPA: hexane/isopropanol; ACE: acetone.

**Table 3:** Pooled within-group correlations between variables and PC functions of the PCA diagram (see Figure 4). For full description of the variables, see Section Material and Methods.

	PC 1	PC 4
Astaxanthin	0.011	−0.010
ME-nd 1	0.014	−0.006
ME-nd 2	0.099	−0.039
ME-C16:2	**0.470**	**−0.129**
ME-nd 3	0.023	−0.065
ME-C18:4	0.038	**0.777**
ME-C16:1	**0.562**	**−0.169**
ME-C18:3	0.166	−0.054
ME-C16:0	**0.595**	0.023
ME-C18:2	0.005	0.039
ME-C20:2	0.051	−0.005
ME-C18:0/C18:1	0.032	0.015
ME-C20:1	0.070	0.016
ME-C20:0	0.030	0.015
ME-nd 4	0.075	0.022
DE-nd 1	−0.010	**−0.126**
DE-C18:4/C18:4	0.093	**0.105**
DE-C18:1/C18:1	0.006	0.005
DE-C18:4/C18:3	0.146	**0.238**
DE-C18:3/C18:3 + DE-C20:4/C22:3	0.010	0.058
DE-C18:1/C18:3	0.128	**0.389**
DE-C26:1/C26:2	0.087	**0.315**
DE-C18:2/C18:3 a	−0.002	0.013
DE-C18:2/C18:3 b	0.000	0.046
DE-nd 2	0.000	0.001
DE-nd 3	0.002	−0.004
**Eigenvalue**	**27.095**	**0.082**
**% variance**	**96.507**	**0.293**

Bold: highlighted values of individual components have a higher correlation contribution.

## Data Availability

Data available upon request from jana.ambrozic@um.si.
